# Impact of air pollution on running performance

**DOI:** 10.1038/s41598-023-28802-x

**Published:** 2023-02-01

**Authors:** Marika Cusick, Sebastian T. Rowland, Nicholas DeFelice

**Affiliations:** 1 Department of Health Policy, Stanford University School of Medicine, Stanford, CA USA; 2 Department of Environmental Health Sciences, Columbia Mailman School of Public Health, New York, NY USA; 3 PSE Healthy Energy, Oakland, CA USA; 4 Department of Environmental Medicine and Public Health, Icahn School of Medicine at Mount Sinai, New York, NY USA; 5 Department of Global Health, Icahn School of Medicine at Mount Sinai, New York, NY USA

**Keywords:** Environmental impact, Climate-change impacts

## Abstract

Air pollution exposures during training may impact race performances. We aggregated data on 334 collegiate male track & field athletes from 46 universities across the United States over 2010–2014. Using distributed lag non-linear models, we analyzed the relationship between race time and PM_2.5_, ozone, and two versions of the Air Quality Index (AQI) exposures up to 21 days prior to the race. We observed a 12.8 (95% CI: 1.3, 24.2) second and 11.5 (95% CI: 0.8, 22.1) second increase in race times from 21 days of PM_2.5_ exposure (10.0 versus 5.0 μg/m^3^) and ozone exposure (54.9 versus 36.9 ppm), respectively. Exposure measured by the two-pollutant threshold (PM_2.5_ and ozone) AQI was not significantly associated with race time; however, the association for summed two-pollutant AQI (PM_2.5_ plus ozone) was similar to associations observed for the individual pollutants (12.4, 95% CI: 1.8, 23.0 s). Training and competing at elevated air pollution levels, even at exposures within AQI’s good-to-moderate classifications, was associated with slower race times. This work provides an initial characterization of the effect of air pollution on running performance and a justification for why coaches should consider approaches to reduce air pollution exposures while training.

## Introduction

High air pollution events such as wildfires have impacted numerous sporting events such as cancellation of several collegiate and professional running races including the Twin Peaks Mile and SF Bay Half Marathon and discussion of relocating a professional National Football League (NFL) game after practice was shortened due to smokey conditions^1^. Exposure to wildfire smoke is experienced throughout the world, creating concerns about whether athletes should train during a period when air pollution levels are high^2^. United States Environmental Protection Agency (USEPA) suggest limiting physical activity during periods of high air pollution^3, 4^; however, little is known about the impact of lower air pollution levels on athletes’ performance or health^2^. These concerns are not limited to wildfires, as there are many other environmental factors, such as vehicle emissions or nearby power plants, that can influence air quality. During exercise, an athlete’s pulmonary tidal volume increases relative to rest^5^ and breathing patterns transition from being nasal to predominantly oral, which bypasses air filtration^6, 7^; with greater airflow velocity, pollutants are carried deeper into the respiratory tract, potentially diffusing into the bloodstream or inflaming the cardiovascular system^8, 9^. Thus, training under moderate air pollution may counteract the benefits of exercise.

Athletic performance suffers in environments with high air pollution^10^. Evidence suggests that the effects of some pollutants are acute; for example, exposure to ozone can immediately impact athletic performance, due to respiratory discomfort^11–13^. Other pollutants like PM_2.5_ (particulate matter smaller than 2.5 microns) have been shown to yield impaired performance up to several days after the high exposure event^14, 15^.To decide when it is safe to train and compete, sports committees such as the National Collegiate Athletic Association (NCAA)^16^ and National Federation of State High School Association (NFHS)^17^ use the Air Quality Index (AQI), a metric developed by the USEPA to communicate air quality risk levels to the public^18^. While these sports committees use the AQI to assess the safety of high pollution events^19–21^, little is done for repeated exposure to moderate levels of air pollution. Recent health studies have found evidence that even repeated low exposure, far below the AQI thresholds of NCAA, has health effects in the general population^22–24^.

Here, we provide a statistically-rigorous assessment of the impact of repeated exposure (i.e., exposure during training) to particulate matter (PM_2.5_) and ozone on race performance of high-caliber track & field collegiate athletes in the United States. We used three different metrics of exposures: (1) pollutant concentrations of PM_2.5_ and ozone, (2) a two-pollutant version of the risk communication index (two-pollutant threshold AQI), and (3) a mixture of exposures by summing together the AQI values (summed two-pollutant AQI) over 21 days prior to their race outcome.

## Methods

### Study design and population

We conducted a retrospective observational study to estimate the association between repeated pollutant exposure and 5-km race times among NCAA collegiate track & field male athletes. To obtain race observations, we used the following procedure: (1) identified universities with top-tier running programs by identifying universities with at least one member who competed in the NCAA Division-1 5-km final race during the years 2010–2014; (2) selected athletes from these universities who competed in identified NCAA sanctioned outdoor track & field 5-km race between March and June during the 2010–2014 NCAA track & field season. The study population was restricted to male subjects due to the time-intensive nature of the manual data collection. Our analysis was exempt from institutional review as all data was publicly available, and our research activity did not involve any interaction with individuals.

### Race observations

Race results and athlete information was obtained from the Track & Field Results Reporting System (TFRRS) database, maintained by the Direct Athletics Incorporation^25^. Details on the database and process of obtaining data are in the supplement (Supplement section A).

### Pollutant exposures

Exposure profiles were developed for the 21 days: 20 training days prior to meet and day of the meet. This exposure profile accounts for the potential cumulative impact of pollutant exposure on cardio-respiratory system during training. Daily air pollution concentrations were assigned to census tracts of each athlete’s home university for 20 days prior to meet and meet location on meet date using the EPA downscaler model^26, 27^. The 21-day period accounts for dates spent training at the home university and competing at races away from the home university. Further details are provided in the supplemental sections B and C.

To calculate the AQI, the EPA designates pollutant-specific concentration breakpoints and provides a linear piecewise function^18^. The AQI breakpoints signify the level of health concern: *good, moderate, unhealthy for sensitive groups, unhealthy, very unhealthy, and hazardous.* When multiple pollutants are measured, the reported AQI is the highest index value among all pollutants. While AQI is traditionally reported based on five pollutants (carbon monoxide, ozone, lead, PM_2.5_, and sulfur dioxide), we calculated the two-pollutant threshold AQI using PM_2.5_ and ozone, which drove 91% of observed AQI values in our study, further analyzed in the supplement section D. Additionally, we hypothesized that the impact of air pollution exposure on performance is not independent between pollutants, so we evaluated the combination of two pollutants as additive rather than substitutionary, defined as the summed two-pollutant AQI value (addition of PM_2.5_-specific and ozone-specific AQI values). We compared the two-pollutant AQI values with the two-pollutant summed AQI values using Kendall’s tau, a measure of correspondence between two measurement approaches.

### Confounders

The meteorological conditions during the meet were measured by matching track race location data to the corresponding grid and time for North American Land Data Assimilation Systems (NLDAS) project-2^28, 29^. The NLDAS data is a 0.125°(~ 13 × 13 km grid cells) gridded product that provides hourly values of temperature measured in Kelvin 2-m above ground (°K), specific humidity measured in kilograms per kilograms 2-m above ground (kg/kg), and 10-m zonal wind speed (m/s) and 10-m meridional wind speed (m/s)—wind speed was calculated as the hypotenuse of these values.

In addition to meteorological variables, we controlled for several performance variables specific to each athlete-race combination. We controlled for the athlete’s personal record prior to the 5-km race being evaluated and the athlete’s previous 5-km race time, both of which represent the athlete’s ability. We also controlled for the number of days since the previous 5-km race, athlete’s year in schooling, and number of days into the calendar year as a proxy for how athletes develop over a season. We included random effects for (a) the athlete’s home university and (b) race.

### Non-linear distributed lag model

We employed distributed-lag non-linear models (DLNMs) to characterize the lagged effects of exposure^30^. DLNMs can capture complex exposure-lag-response relationships by simultaneously adjusting for exposure at each lag, via non-linear terms such as natural splines. This type of model has been used in other studies on the lagged effects of air pollution and temperature on health outcomes^31–36^. In a DLNM, lagged exposure is represented as a crossbasis term, a combined basis matrix for the exposure dimension and lag dimension. For each exposure, the constraints of its basis matrix, i.e., its functional form, were chosen via Akaike information criterion (AIC). We tested varying degrees of freedom (df = 3, 4, 5) and equal and logarithmic lag placements for both exposure and lag dimensions.

### Final model

We estimated the association between air pollutant exposure and race times using mixed-effects linear models. First, we used the backwards stepwise AIC function to select the covariates of the base mixed-effects linear model without the crossbasis terms for air pollution exposure. Next, for each of the four exposures, we ran the model with selected covariates and compared AIC values, while varying functional forms of the cross-basis matrix. Our final model is represented by the following Eq. (1).

1 $${E[O}_{i,j,k}] =\sum_{l=1}^{21}{\beta }_{l}{T}_{l}+{{\varvec{\lambda}}X}_{i}+{\delta }_{i}+{\gamma }_{k}$$

In this equation, *i, j, k* represent the race, athlete, and athlete’s home university of interest, respectively; ${O}_{ij}$ represents the 5-km race outcome of interest in total altitude-adjusted seconds; ${T}_{l}$ is the air pollution exposure matrix obtained by applying the basis functions to either PM_2.5,_ ozone, two-pollutant threshold AQI, or summed two-pollutant AQI; ${\beta }_{l}$ represents the coefficients for the lagged air pollution exposure matrix ${T}_{l}$ differentiated by the lag day (*l*), which ranges from 0 to 21 days. For each of our four exposures, according to the AIC, we identified the optimal form of the cross-basis matrix for both the exposure–response and lag-response functions to be a natural cubic spline with 5 degrees of freedom (df) at equally spaced knots. The covariates in our equation are represented by ${X}_{i}$, and ${\varvec{\lambda}}$ is the vector of coefficients. Random intercepts for both the race and the athlete’s home university are represented by ${\delta }_{i}$ and ${\gamma }_{k},$ respectively. In the final model, the backwards AIC algorithm selected all covariates except for the previous 5-km race time. We estimated lag-response relationships and cumulative effect estimates for comparing exposure at the 80th percentile with the 20th percentile.

All analyses were conducted using R version 4.0.3 and the following packages: dlnm version 2.4.6 for the distributed lag model, lme4 version 1.1–27.1 for linear mixed-effects models, and MLmetrics version 1.1.1 for evaluation metrics^30, 37, 38^. All included data are publicly available; data and code used for analysis is available on Github (https://github.com/marikamaecusick/RunningAP).

### Sensitivity analyses

First, given uncertainty of the impact that different lagged exposures may impose, we considered alternative number of lags by running the models using 14-day and 28-day lags for all four of our exposures. Second, we fit 1000 negative exposure models, which evaluate the impact of a perturbed exposure matrix randomly sampled from exposures in our study, on race outcomes. If we detected statistically significant associations for more than 5% of the models, this would suggest that our confidence intervals are overly confident (i.e., too narrow)^39, 40^. We assessed the percentage of perturbed exposure matrices that had a statistically significant cumulative effect for each of our exposures when comparing the 20–80th percentile exposure.

## Results

Summary statistics of the NCAA Division I Outdoor Track & Field 5-km race observations included in our study are presented in Table 1. After eliminating race results with missing information (n = 604), we identified a total of 1,104 performances at 143 races run by 334 elite male collegiate athletes from 46 different universities. The average altitude-adjusted race time was 14 min: 17 s (857.3 s) with a standard deviation of 26.2 s, and race times ranged from 13 min: 15 s (795 s) to 16 min: 9 s (969 s).

**Table 1:** Descriptive statistics.

Variable	Count
*Performance Athlete School Year*	
Freshman	101
Sophomore	257
Junior	355
Senior	391

	Mean (SD) [minutes:seconds]
Previous 5-km Time (s)	855.9 (26.5) [14:15.9]
5-km Personal Record (s)	844.5 (26.3) [14:04.5]
Days since Previous 5-km	20.4 (11.0)
Days into the year	131.7 (19.8) (5/11)
Race Temperature (C)	16.8 (7.1)
Race Specific Humidity (kg/kg)	8.7 (3.8)
Race Wind (m/s)	3.7 (1.8)

EPA regions*	Number of athletes
Region 1—Boston (serving CT, ME, MA, NH, RI, and VT)	23
Region 2—New York City (serving NJ, NY, Puerto Rico, the U.S. Virgin Islands and 8 federally recognized Indian Nations)	52
Region 3—Philadelphia (serving DE, DC, MD, PA, VA, WV and 7 federally recognized tribes)	57
Region 4—Atlanta (serving AL, FL, GA, KY, MS, NC, SC, and TN)	40
Region 5—Chicago (serving IL, IN, MI, MN, OH, and WI)	49
Region 6—Dallas (serving AR, LA, NM, OK, and TX)	38
Region 7—Kansas City (serving IA, KS, MO, and NE)	4
Region 8—Denver (serving CO, MT, ND, SD, UT, and WY)	17
Region 9—San Francisco (serving AZ, CA, HI, NV, American Samoa, Commonwealth of the Northern Mariana Islands, Federated States of Micronesia, Guam, Marshall Islands, and Republic of Palau)	40
Region 10—Seattle (serving AK, ID, OR, WA and 271 native tribes)	20

*6 athletes transferred to another region within study period.

### PM_2.5_ and ozone concentrations

**Figure 1:**
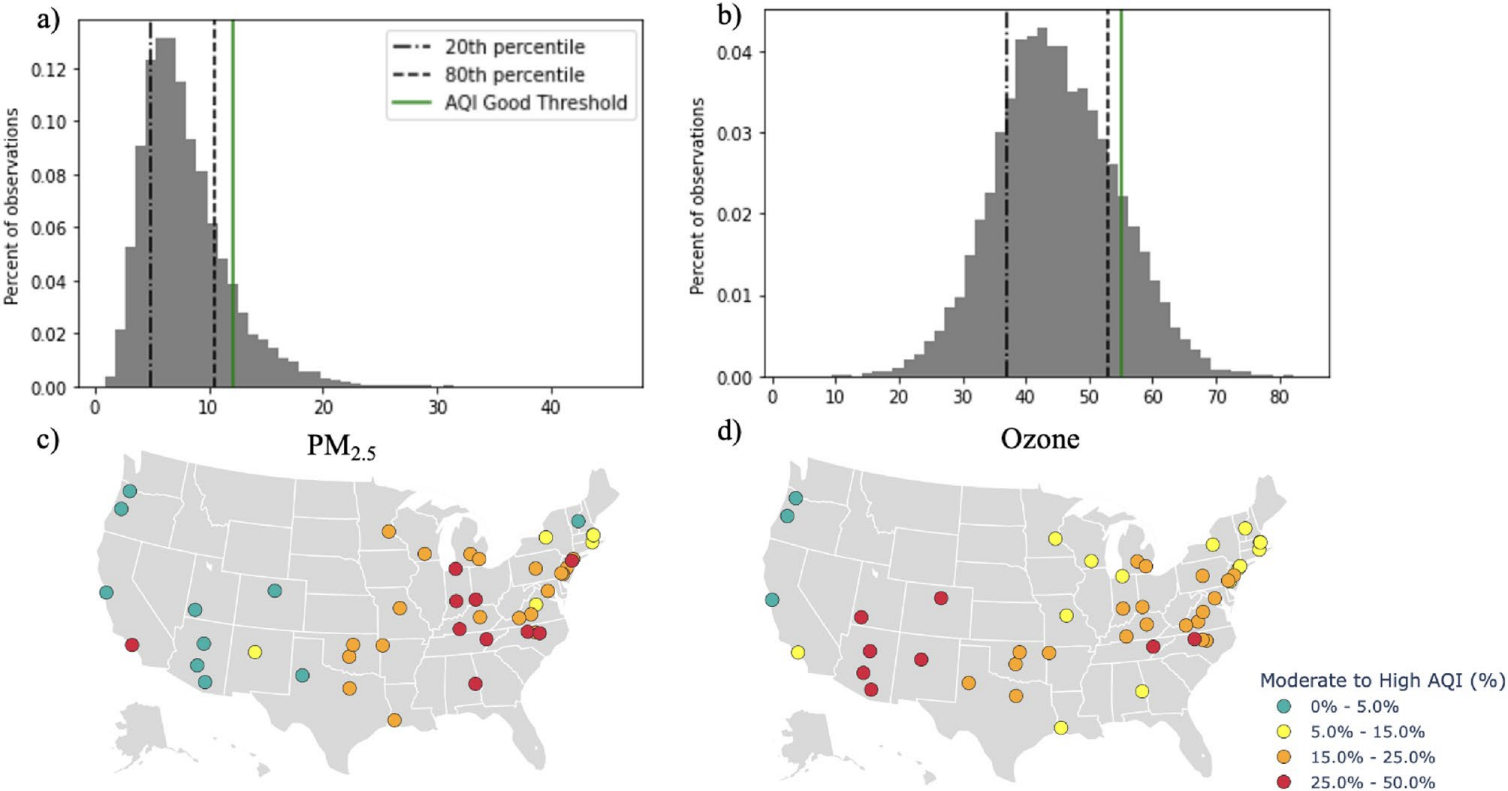
(**a**)–(**d**) Variations in PM_2.5_ and ozone concentrations (**a**) histogram of PM_2.5_ concentrations, the dashed line, dotted line and green line represent the 20th percentile: 4.9 $\mathrm{\mu g}/{\mathrm{m}}^{3},$ 80th percentile: 10.3 $\mathrm{\mu g}/{\mathrm{m}}^{3}$ and AQI *good* threshold (< 50): 12 $\mathrm{\mu g}/{\mathrm{m}}^{3}$, respectively (**b**) histogram of ozone concentrations, the dashed line, dotted line, and green line represent the 20th percentile: 36.9 ppm, 80th percentile: 54.9 ppm, and AQI *good* threshold (< 50): 54 ppm, respectively (**c**) map of university locations colored by percentage of days with moderate-to-high PM_2.5_ according to PM_2.5_-specific AQI (**d**) map of university locations colored by percentage of days with moderate-to-high ozone according to ozone-specific AQI. Software: python version 3.9.1, matplotlib version 3.3.3 (histograms 1a, b), plotly version 5.5.0 (maps 1c, d).

Figure 1a,b illustrate the distribution of observed pollutant (PM_2.5_ and ozone) exposures. 88% were within the *good* category for PM_2.5_ (0–12.0 $$\upmu {\text{g}}/{\text{m}}^{3}$$), 12% were within the *moderate* category (12.1–35.4 $$\upmu {\text{g}}/{\text{m}}^{3}$$), and less than 1% were within the *unhealthy for sensitive groups* category (35.5- 55.4 $$\upmu {\text{g}}/{\text{m}}^{3}$$$$)$$ (Fig. 1a). Of the ozone exposures in our study, 79% were within the *good* category (0–54 ppm), 20% were within the *moderate* category (55–70 ppm), and less than 1% were within the *unhealthy for sensitive groups* category (71–85 ppm) (Fig. 1b).

Figure 1c,d (maps) illustrate the variability of exposure across home university locations. PM_2.5_ and ozone exposures differ across university locations, as evident by the percentage of days with pollutant exposures higher than AQI’s pollutant-specific *good* threshold during the study period.

### Associations of PM_2.5_ and ozone

**Figure 2:**
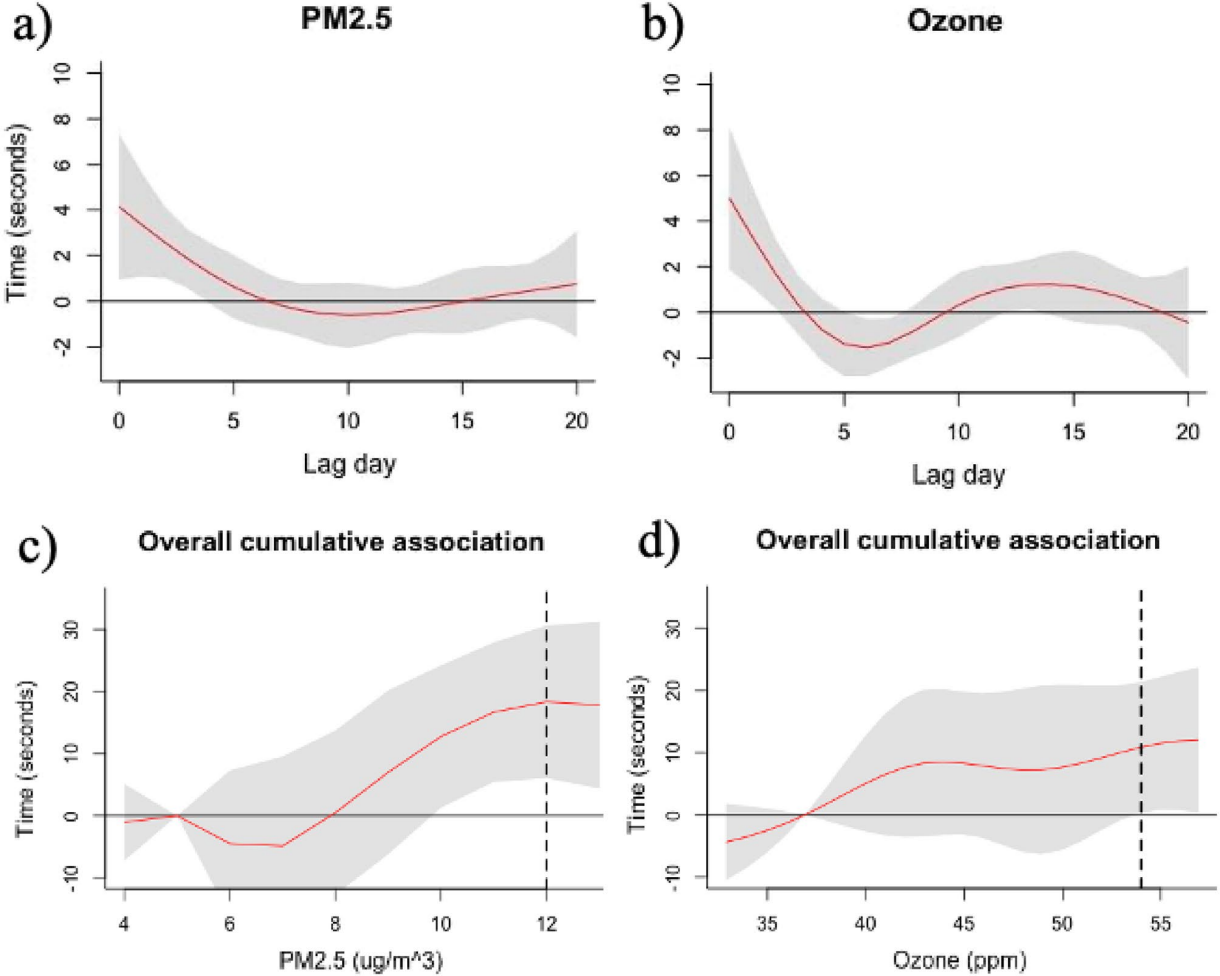
PM_2.5_ (**a**) and ozone (**b**) lag-response relationship when comparing our reference levels: 20th percentile exposure with 80th percentile exposure over a 21-day training period (red line) with 95% confidence intervals (grey). Cumulative association of PM_2.5_ (**c**) and ozone (**d**) on race performance (seconds) over the 21-day training period (red line) with 95% confidence intervals (grey) and AQI *good* threshold (dashed black line). Software: R version 4.0.3 dlnm version 2.4.7.

We observed a cumulative 12.8 (95% CI: 1.29, 24.2) second increase in 5-km race times from 21 days of exposure to the PM_2.5_ concentration at the 20th percentile (5.$0 \upmu \mathrm{g}/{\mathrm{m}}^{3}$, AQI = 20) compared to the 80th percentile (10.$0 \upmu \mathrm{g}/{\mathrm{m}}^{3}$, AQI = 43) (Fig. 2a). Cumulative PM_2.5_ exposure at levels higher than 8 $\mathrm{\upmu g}/{\mathrm{m}}^{3}$ were associated with higher overall race times, and associations were statistically significant at levels higher than 10 $\upmu \mathrm{g}/{\mathrm{m}}^{3}$ but below AQI’s *good* threshold (Fig. 2c).

There was a cumulative 11.5 (95% CI: 0.8, 22.1) second increase in 5-km race time during the 21-day period of exposure when comparing the 20th percentile (36.9 ppm, AQI = 33) with the 80^th^ percentile (54.9 ppm, AQI = 50) (Fig. 2b). We observed a positive cumulative association between ozone exposure and race time increases at a range of levels higher than 36 ppm, yet associations were only statistically significant above the AQI *good* threshold (54 ppm) (Fig. 2d).

### Associations of two-pollutant threshold AQI and summed two-pollutant AQI

**Figure 3:**
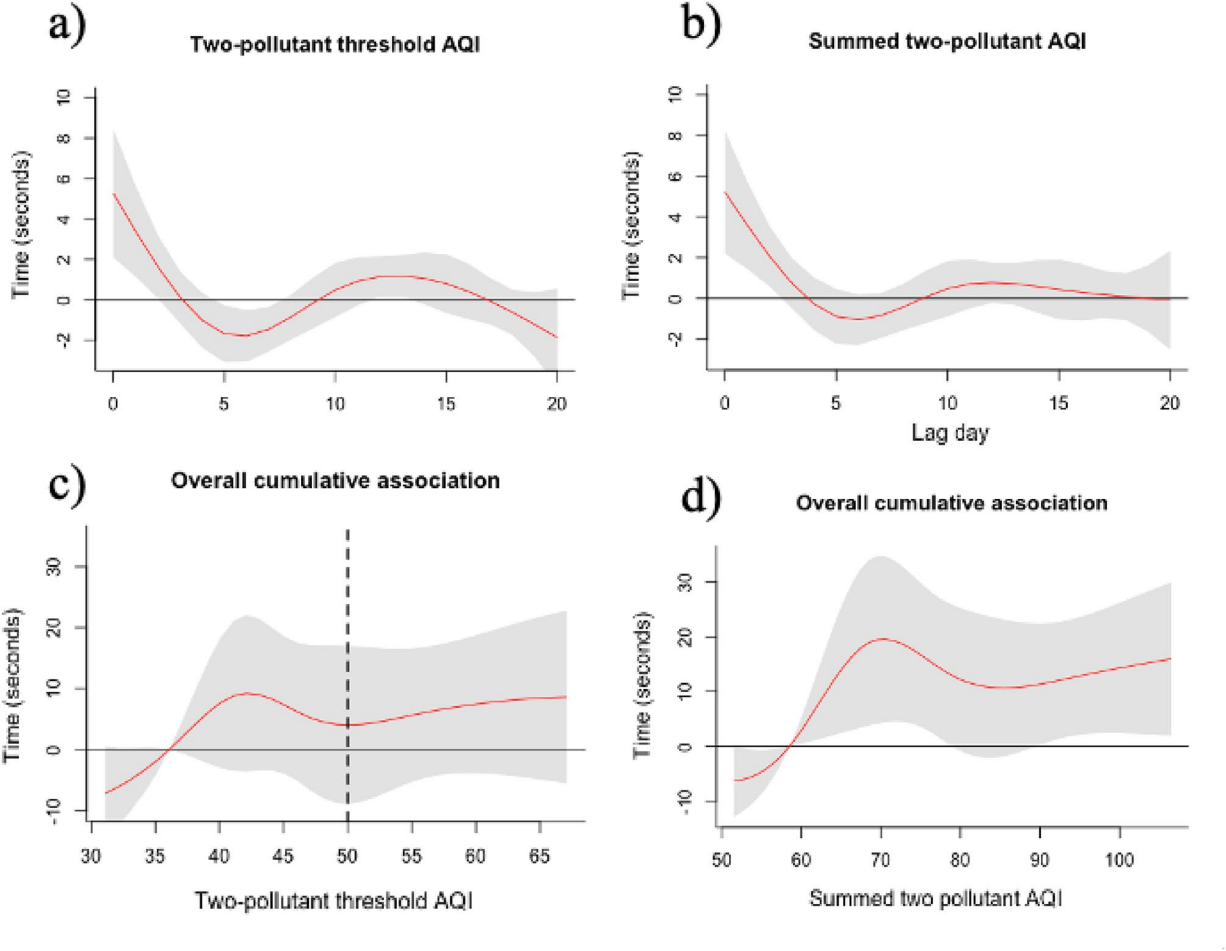
Two-pollutant threshold AQI (**a**) and summed two-pollutant AQI (**b**) lag-response relationship when comparing our reference levels: 20th percentile exposure with 80th percentile exposure over a 21-day training period (red line) with 95% confidence intervals (grey). Cumulative association of two-pollutant threshold AQI (**c**) and summed two-pollutant AQI (**d**) on race performance (seconds) over the 21-day training period (red line) with 95% confidence intervals (grey) and AQI *good* threshold (dashed black line). Software: R version 4.0.3 dlnm version 2.4.7.

In comparing the two AQI decision indices using the Kendall-Tau measure, we reject the null hypothesis that the two indices are statistically independent with a statistic of 0.68 and a p-value of 0.0. Cumulative exposure over the 21-day period measured by the two-pollutant threshold AQI was not significantly associated with race time (95% CI: − 5.23, 16.60) when comparing the 20th percentile (AQI = 36.1) with the 80th percentile (AQI = 55.1) (Fig. 3a). This result is emphasized by the Fig. 3c—while increases in the two-pollutant threshold AQI are associated with higher race times (Fig. 3d), there is no value at which the cumulative association is statistically significant at the 95% confidence level.

The cumulative association of increased exposure at the 80th percentile (93.5) vs. the 20th percentile (58.5) measured by the summed two-pollutant AQI was similar to that seen of the individual pollutants (12.4 s, 95% CI: 1.76, 23.03) (Fig. 3b). We observed an increasing cumulative association between summed two-pollutant AQI exposure and slower race times at levels higher than 60.

### Sensitivity analyses

Using a thousand iterations of the perturbed exposure values, we observed that less than 5% had statistically significant cumulative associations for each of our exposures (PM_2.5_, ozone, two-pollutant threshold AQI, and summed two-pollutant AQI) when comparing our reference levels, 20th percentile exposure with 80th percentile. Additionally, less than 2% of the perturbed exposures simulations had statistically significant cumulative increases in race times, thus providing more confidence that this is a valid model.

Cumulative effect estimates after using 14-day and 28-day lags were consistent with those of the 21-day lags for all our exposures (see supplementary Table 1, Figs. S7–S10). Lag-response relationships remained like the 21-day lag relationships displayed in Figs. 2 and 3 (see supplementary Fig. S3–7).

## Discussion

This study estimated the association of repeated air pollution exposure, experienced during training and competition, with race times of high-caliber male collegiate athletes. Our DLNM models determined that athletes who experienced higher levels of PM_2.5_ and ozone during their 21-day training cycle prior to the race were associated a slower 5-km race time than expected. Specifically, for PM_2.5_ and ozone respectively, comparing 21 days of exposure at 80th percentile and 20th percentile, was associated with 1.5% (12.8 s) and 1.3% (11.5 s) increases in the average race time observed in our study. While these increases may seem small, in races as competitive as the NCAA Division I 5 km championships, a 12 second increase can differentiate between 1st and 6th place or separate those selected to be on the All-American team.

While repeated exposure measured by the two-pollutant threshold AQI was not associated with a statistically significant cumulative impact on race times, exposure measured by the summed two-pollutant AQI (PM_2.5_ plus ozone) was significant. While the Kendall rank correlation coefficient suggests the two decision indices are dependent, our results indicate differential impact of repeated exposure, suggesting these indices still capture athletes’ exposure profile differently. The two-pollutant threshold AQI, which takes the maximum of each pollutant’s AQI value, may underestimate the athlete’s exposure profile.

### Observed pollutant exposures

Overall, athletes in our study were training and competing in relatively good air quality conditions. No university included in our study had more than 50% of days above the *moderate* threshold, with the largest percentage of days at a single university being 37.2% and 49.8% for PM_2.5_ and ozone, respectively. We observed significant associations with race performances for air pollution increases from the 20–80th percentile; for both pollutants even the 80^th^ percentile was within the AQI's *good* threshold (Fig. 1), indicating even increases in air pollution under good air quality conditions may affect performance.

### Previous literature

Results from our analysis align with previous studies on the association between air pollution exposure (PM and ozone) on athletic performance^10^. Prior research has focused mostly on the immediate impacts of hazardous levels of PM and ozone among healthy young adults and athletes in controlled experiments (e.g. results of maximal exercise tests). Most, but not all of these studies found that acute exposures to high levels of PM (PM_1_: 336,700–396,200 particles/cm^3^) and ozone (120–350 ppb) impair the maximal accumulated work on short exercise tests^41–44^. In the observational context, studies have demonstrated increased air pollution to be associated slower marathon race times^45–48^.

Literature on the effect of repeated exposures and the timing of these impacts is more limited, particularly in the observational context. Healthy athletes exposed to high levels of PM in controlled experiments (ergometer trials at PM_1_ 396,200 particles/cm^3^) and natural environments (urban cities with mean levels of PM_2.5_ at 65.1 $\upmu \mathrm{g}/{\mathrm{m}}^{3}$) experienced significant impairments in performance several days later, suggesting delayed inflammatory effects^14, 15^. However, some conflicting evidence exists, as male cyclists with 60 min of exposure to 300 $\upmu \mathrm{g}/{\mathrm{m}}^{3}$ of PM_2.5_ immediately prior to exercise had no impairments on their time trial^49^. Similar studies on the effect of high ozone environments (controlled: 0.12–0.24 ppm and natural: average: 33.96 ppb) found that athletes experienced immediate performance reductions, yet there was a protective effect of pre-race ozone exposure^50, 51^.

Our analysis adds to the air pollution and athletic performance literature as we used a natural experiment design rather than controlled lab-based exposures to estimate the association of repeated exposures at lower, less hazardous levels for two different pollutants and provided context for decision-makers who rely on a commonly used decision index (AQI) rather than raw pollutant concentrations.

### Implications for the athletic community

Our analysis has implications for the broader athletic community. Athletes included in our study were among the fittest in the general population, and yet, adverse impacts on athletic performance was observed. The association may be more pronounced among more sensitive athletes, such as young athletes still in physical development or those with underlying conditions, such as asthma^52, 53^. Thus, when coaches make decisions about their athletes’ training and competition schedules, it may be important to also factor in pollutant exposures by understanding pollutant levels within the area and tracking seasonal and daily trends of exposures levels and provide appropriate recovery between training sessions.

## Limitations

First, we focused on PM_2.5_ and ozone, and the three other critical air pollutants, carbon monoxide, nitrogen dioxide, and sulfur dioxide, may also have impacted race performance. We made this choice for two primary reasons: first, these pollutants are the most prevalent in the United States (evidenced by the fact that 91% of the AQIs were determined from PM_2.5_ or ozone), and second, there was a large body of literature on the adverse effects of these pollutants on athletic performance^5, 10–12, 14, 15, 54^. Second, because not all locations in our study had a nearby EPA monitor, we relied on outputs from the downscaler model, which extrapolates exposure according to the closest monitoring station^26, 27^. Third, exact location of athlete’s whereabouts during the track & field seasons were unable to be captured, leading us to possibly over- or underestimated exposure. Finally, our results have limited transportability, as we examined only male athletes competing in a single track & field event. Thus, our results can be viewed as an initial finding indicating the importance of this relationship, though the magnitude of effect likely varies in athletes outside of this study population. Future research into this topic should include female athletes, as well as athletes of any gender, other age ranges and level of fitness, as well as consider other athletic events, to understand impacts of air pollution in other populations and other contexts.

## Conclusion

This retrospective study quantified the association between repeated exposure to air pollution and athletic running performance. Training and competing at consistently higher levels of air pollution, even below the EPA’s threshold for *good* air quality and among high-caliber athletes, was associated with slower race times. This first step at identifying adverse effects of repeated exposure to air pollution provides a foundation for why coaches should consider approaches to minimize exposure among their athletes.

## Supplementary Information

Supplementary Information.

## Data Availability

Datasets and code used for analysis are publicly available at https://github.com/marikamaecusick/RunningAP.
