# Supplementary material for: Impact of air pollution on running performance
Source: Sci Rep. 2023 Feb 1;13:1832. doi: 10.1038/s41598-023-28802-x (PMC9892497; doi:10.1038/s41598-023-28802-x)
Supplement: Supplementary file 1 — Supplementary Information. [file 41598_2023_28802_MOESM1_ESM.docx]

**SUPPLEMENT**

**A. Race observations**

The Track & Field Results Reporting System (TFFRS) database serves as a mandatory collegiate track & field results reporting clearinghouse and statistics engine for almost every 4-year and 2-year academic institution in the United States. Official results are uploaded electronically from the timing systems used at the race, automatically updating race results, athlete profiles, and rankings. Using the python BeautifulSoup package(44), we scraped athletes’ TFRRS profiles, obtaining information on each of their races, including race locations, race dates, race times, and placement. We obtained the specific date and time of the 5-km race through a manual review of meet schedules from collegiate websites. For races that took place at altitudes over 3,000 feet, we adjusted race times to match sea-level expected performance using the NCAA race time conversion executable found on the U.S. Track & Field and Cross Country Coaches Association (USTFCCCA) website (45).

**B. Pollutant exposures**

The United States Environmental Protection Agency (EPA) has developed a Bayesian space-time downscaler model that fuses observations from the National Air Monitoring Stations/State and Local Air Monitoring observations (NAMS/SLAMS) with a 12km gridded-output from the Community Multiscale Air Quality (CMAQ) mode to generate a daily ozone (8-hr max) and PM_2.5_ (24-hr average) estimate (27,28). Pollutant concentrations are reported at the census tract level according to the latitude and longitude of the tract’s centroid.

After obtaining the latitude and longitude for each location of interest (universities and race locations) using the Google Geocoding API (27), we identified the nearest census tract centroids reported in the EPA downscaler model. Using these census tracts, we obtained daily PM_2.5_ and ozone measurements to assign a 21-day exposure for each athlete prior to their 2010-2014 race.

**C. Exposure matrix construction**

For each exposure of interest, we identified the exposure values for three weeks at the athlete’s home university location prior to the race outcome. If the race took place at a location more than 100 miles away from the home university, we assumed that the athlete travelled to the meet location one day prior to the race, and thus was exposed to the pollutant levels of the meet location for two days. Otherwise, we assumed that the athlete arrived at the meet location on the day of the race, and thus was only exposed for one day. For athletes that had ran other meets during the 21-day period prior to the race, we applied the same logic to ensure their pollutant exposures reflected their training and competition locations.

**D. Validation of the two-pollutant threshold AQI**

To validate that the two-pollutant threshold AQI values in this study were roughly equivalent to the traditional five-pollutant AQI, we conducted an analysis of AQI values across the United States during the months March – June of 2010-2014 to determine that 65% of the values were attributed to ozone, 26% of the values were attributed to PM2.5, and the remaining 9% were attributed to the remaining three pollutants in the following order: sulfur dioxide, lead, and carbon monoxide.

**E. Lag-response relationship analysis**

As shown in Figure 2A, increased exposure to PM_2.5_ at the 80^th^ percentile (10.3 $\mu g/m^{3}$) in comparison to the 20^th^ percentile (4.9$\mu g/m^{3}$) was associated with slower race times during the four days leading up to the race, association on race day was 4.1 seconds (95% CI: 0.94, 7.33).

As seen in Figure 2B, increased exposure to ozone at the 80^th^ percentile (54.9ppm) in comparison to the 20^th^ percentile (36.9ppm) was significantly associated with slower race times during the two days leading up to the race and with faster race times by 1.55 (95% CI: -0.29, -2.81) seconds on the seventh day prior to the race.

As shown in figure 3A, increased exposure measured by the two-pollutant threshold AQI at the 80^th^ percentile (55.1) in comparison to the 20^th^ percentile (36.1) was associated with statistically significant slower race times in the three days leading up to the race, with the first day having an effect of 5.3 seconds (95% CI: 2.1, 8.5). However, like the ozone lag-response relationship shown in Figure 2, the exposure was associated with faster race times on the sixth day by 1.7 seconds (95% CI: -0.3, -3.1) prior to the race.

As shown in figure 3B, increased exposure measured by the summed two-pollutant AQI at the 80^th^ percentile (93.5) in comparison to the 20^th^ percentile (58.5) was associated with slower race times with statistical significance during the three days leading up to the race, 5.2 seconds (95% CI: 2.2, 8.2) on race day, similar to the PM_2.5_ lag-response relationship shown in Figure 2. Increased exposure measured via two-pollutant threshold AQI and summed two-pollutant AQI more than one week from the race did not have statistically significant effects.

**F. Variability of observed race times**

An addition or subtraction of 12 seconds from the average race time in our study captures 35% of all race times in our study (Supplement Figure 1), and an addition or subtraction of 12 seconds from the average NCAA championship race time captures 47% of the NCAA championship races times (Supplement Figure 2).


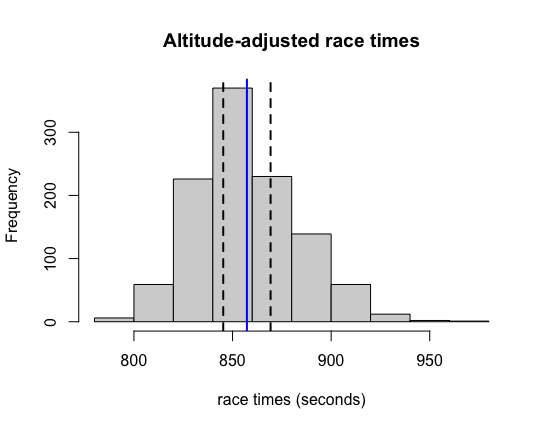


**Supplement Figure 1. Histogram of altitude-adjusted race times with the mean race time (blue line) plus/minus 12 seconds (dashed black lines), N=1,104.**


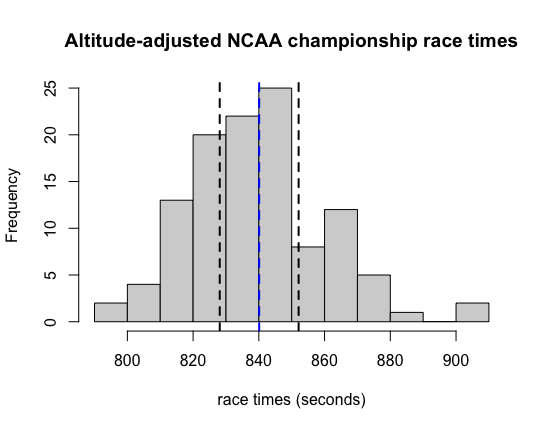


**Supplement Figure 2. Histogram of altitude-adjusted NCAA Division 1 championship race times with the mean race time (blue line) plus/minus 12 seconds (dashed black lines), N=114.**

**Supplement Table 1. Pollutant concentration cumulative effect on race time (21-day, 14-day, and 28-day lags)**

| Exposure | Baseline | Comparison | Time effect (s) (95% CI)  21 days | Time effect (s) (95% CI)  14 days | Time effect (s) (95% CI)  28 days |
| --- | --- | --- | --- | --- | --- |
| PM_2.5_ | 4.9$\mu g/m^{3}$ | 10.3$\mu g/m^{3}$ | 12.8 (1.3, 24.2) | 11.0 (0.3, 12.6) | 11.4 (-0.9, 23.78) |
| Ozone | 36.9 ppm | 54.9 ppm | 11.5 (0.8, 22.1) | 8.64 (-1.6, 18.9) | 11.1 (0.0, 22.2) |
| Two-pollutant threshold AQI | 36.1 | 55.1 | 5.7 (-5.2, 16.6) | 5.15 (-5.48, 15.77) | 5.08 (-6.20, 16.35) |
| Summed two-pollutant AQI | 58.5 | 93.5 | 12.4 (1.8, 23.0) | 11.3 (1.2, 21.4) | 9.4 (-2.5, 21.2) |


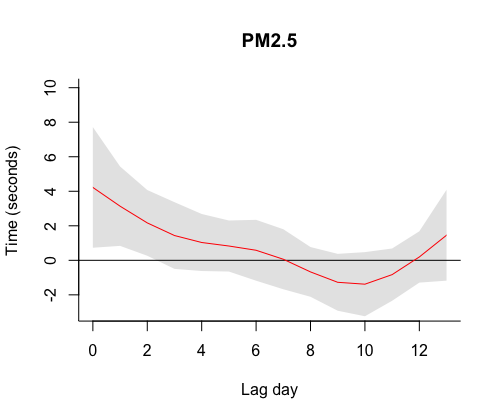

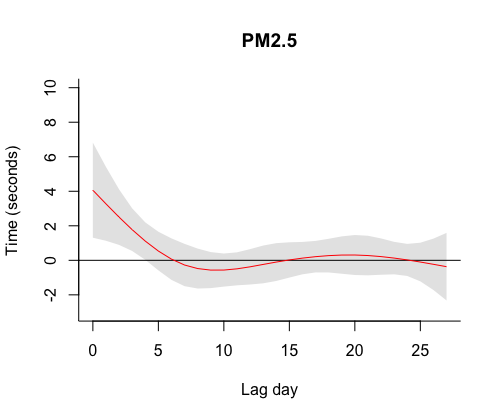
**Supplement Figure 3. PM_2.5_ lag-response relationship when comparing 80^th^ percentile exposure with 20^th^ percentile exposure over a 14-day (left) and 28-day (right) training period (red line) with 95% confidence intervals (grey).**


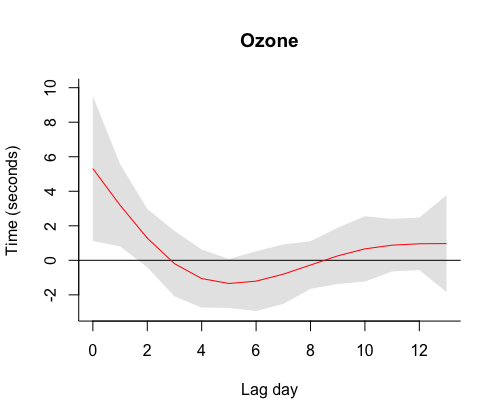

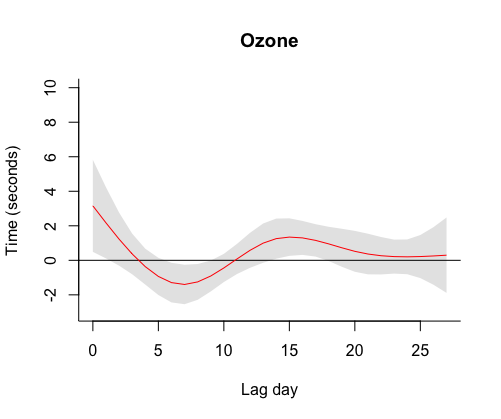


**Supplement Figure 4. Ozone lag-response relationship when comparing 80^th^ percentile exposure with 20^th^ percentile exposure over a 14-day (left) and 28-day (right) training period (red line) with 95% confidence intervals (grey)**


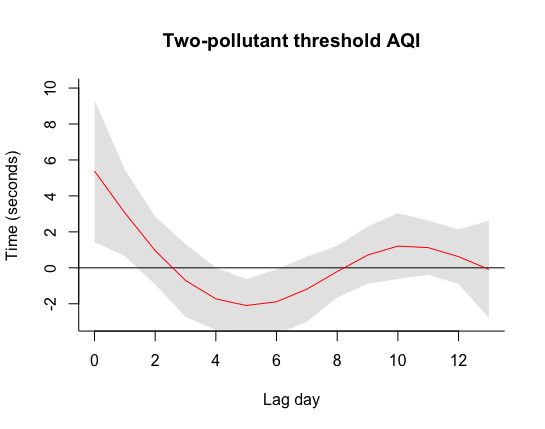

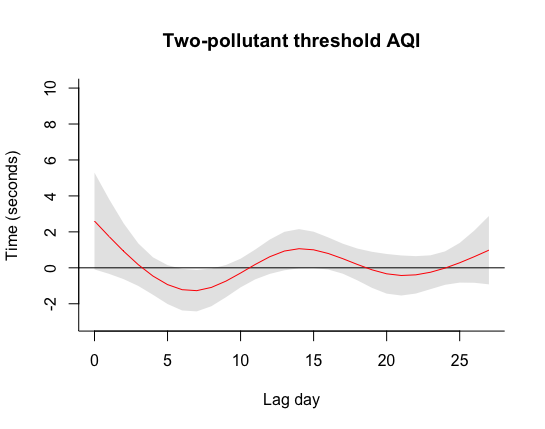


**Supplement Figure 5. Two-pollutant threshold AQI lag-response relationship when comparing 80^th^ percentile exposure with 20^th^ percentile exposure over a 14-day (left) and 28-day (right) training period (red line) with 95% confidence intervals (grey)**


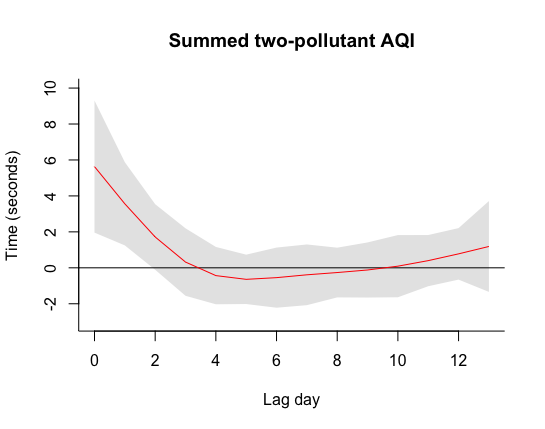

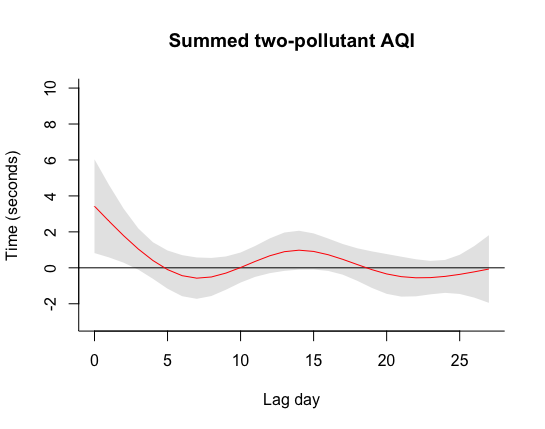


**Supplement Figure 6. Summed two-pollutant AQI lag-response relationship when comparing 80^th^ percentile exposure with 20^th^ percentile exposure over a 14-day (left) and 28-day (right) training period (red line) with 95% confidence intervals (grey)**


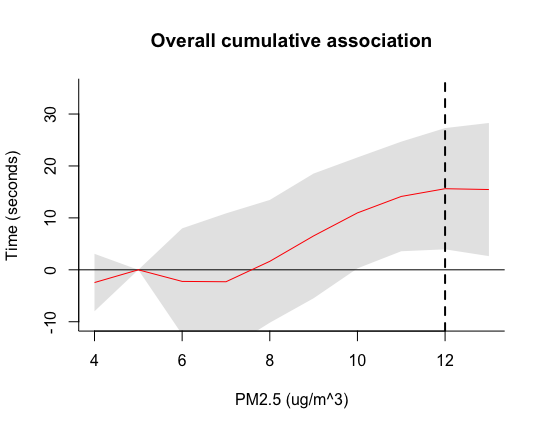

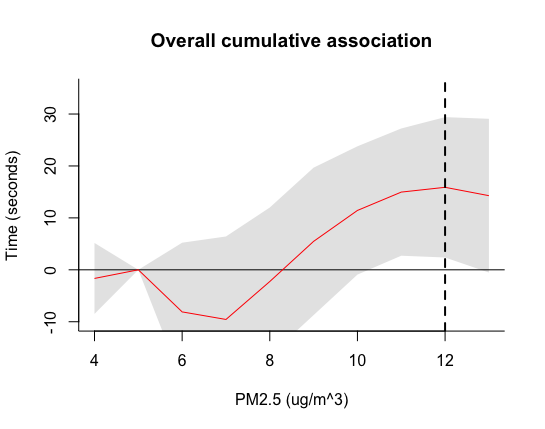


**Supplement Figure 7. Cumulative association of PM_2.5_ on race performance (seconds) over the 14-day (left) and 28-day (right) training period (red line) with 95% confidence intervals (grey) and AQI *good* threshold (dashed black line)**

_
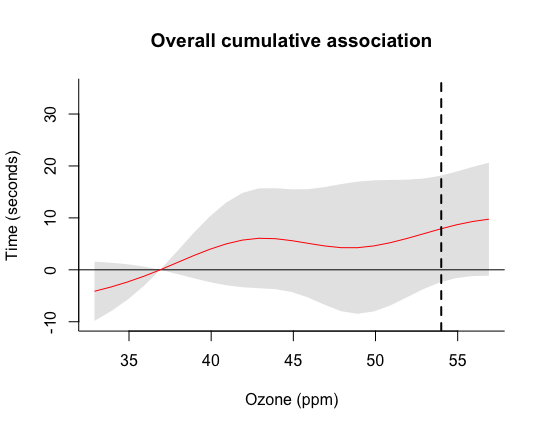

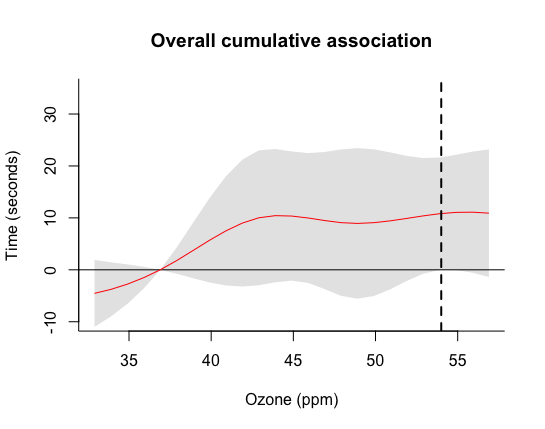
_

**Supplement Figure 8. Cumulative association of ozone on race performance (seconds) over the 14-day and 28-day training period (red line) with 95% confidence intervals (grey) and AQI *good* threshold (dashed black line)**


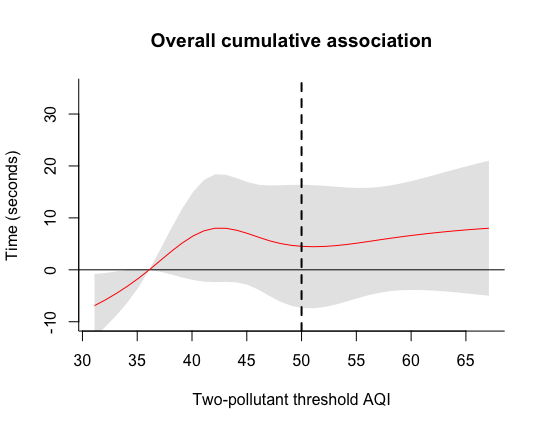

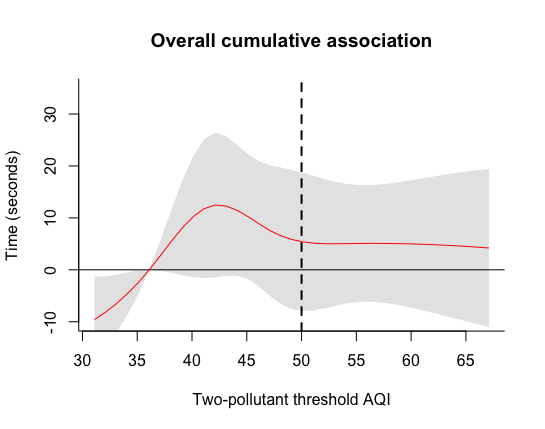


**Supplement Figure 9. Cumulative association of two-pollutant threshold AQI on race performance (seconds) over the 14-day (left) and 28-day (right) training period (red line) with 95% confidence intervals (grey) and AQI *good* threshold (dashed black line)**


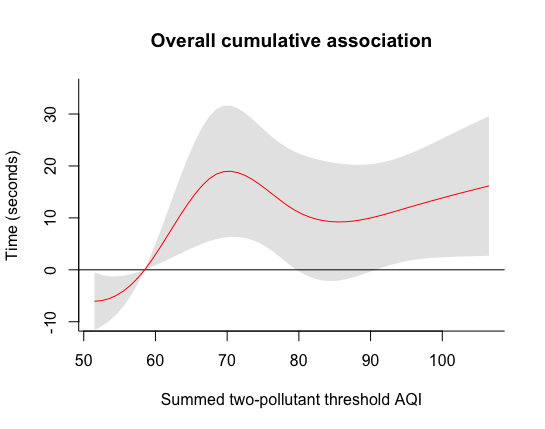

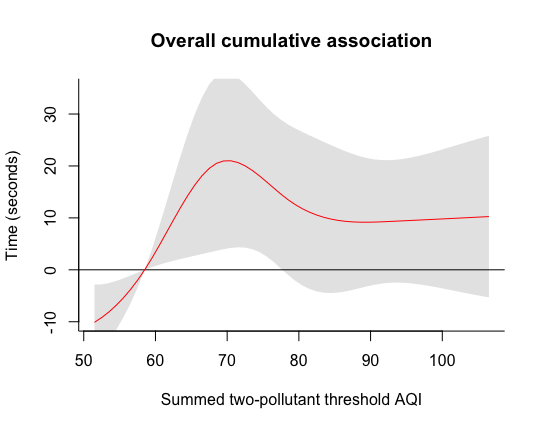


**Supplement Figure 10. Cumulative association of summed two-pollutant AQI on race performance (seconds) over the 14-day (left) and 28-day (right) training period (red line) with 95% confidence intervals (grey) and AQI *good* threshold (dashed black line)**
